# Prevalence, knowledge, and concerns regarding the use of heated tobacco products and electronic cigarettes among young Japanese physicians

**DOI:** 10.18332/tid/178508

**Published:** 2024-02-16

**Authors:** Yuichiro Otsuka, Yoshitaka Kaneita, Osamu Itani, Suguru Nakajima

**Affiliations:** 1 Division of Public Health, Department of Social Medicine, Nihon University School of Medicine, Tokyo, Japan

**Keywords:** heated tobacco products, e-cigarettes, Japan, physician, smoking status

## Abstract

**INTRODUCTION:**

Heated tobacco products (HTPs) and e-cigarettes (ECs) have gained traction as alternatives for harm reduction, especially in Japan. In particular, the use of HTPs is rapidly gaining popularity among young adults in Japan, with a prevalence of 10.9% in 2020. Despite uncertainties regarding the health effects of HTPs and ECs, concerns regarding nicotine and carcinogens persist. Although physicians play a vital role in smoking cessation, they lack awareness and concerns regarding HTPs. This study aimed to assess the prevalence, knowledge, and concerns regarding HTPs and ECs among young Japanese physicians.

**METHODS:**

A cross-sectional online survey was conducted in 2021 with 529 young Japanese physicians aged 24–39 years. Parameters assessed included awareness, smoking status, knowledge of HTPs and ECs, and concerns related to HTPs. Statistical analyses were conducted to assess prevalence, knowledge, and concerns by smoking status using the chi-squared test and logistic regression.

**RESULTS:**

Most participants were aware of HTPs (89.0%) and ECs (71.3%). Young male physicians preferred HTPs, while females favored ECs. Primary sources of information included newspapers and stories (56.8%), and TV (37.4%). Non-smokers (89.0%) demonstrated limited knowledge of these products. Concerns were highest and lowest among non-smokers and HTP users, respectively, with safety concerns being the most prevalent.

**CONCLUSIONS:**

Young physicians exhibited lower smoking rates than the general population, but HTP use was prominent among young male physicians. Concerns varied based on smoking status, indicating the need to address these issues among healthcare professionals. Despite high awareness, knowledge gaps, particularly among non-smokers, highlight the importance of public health and educational campaigns to disseminate knowledge among physicians, regardless of medical specialty.

## INTRODUCTION

The tobacco industry has been promoting ‘harm reduction’ through heated tobacco products (HTPs) in recent years^1^, similar to electronic cigarettes (ECs)^2^. These products are marketed as technological innovations and socially acceptable alternatives to conventional cigarettes (CCs)^1,2^. HTPs were first introduced in Japan and gained substantial global popularity in over 50 countries^3^. Their current prevalence surged from 0.2% in 2015 to 10.9% in 2020 among individuals in Japan aged 15–74 years^4^. In a nationwide study, the prevalence of current HTP smokers was 8.3% among males and 1.9% among females^5^. Remarkably, their popularity was particularly pronounced among younger individuals, specifically those aged 20–39 years^4^. A cross-sectional study in Korea revealed that certain smokers opted for HTPs as a smoking cessation aid^6^.

However, the health ramifications of HTP and EC use remain uncertain. Consequently, public health researchers have asserted that a substantial number of these products continue to pose health hazards, affecting both smokers and non-smokers^7,8^. This predicament was primarily attributable to the presence of nicotine, carcinogenic agents, and considerable metal emissions in the exhaled vapor^7,9^.

Physicians, held as paragons of health behavior, shoulder a pivotal responsibility in administering guidance for smoking cessation^10,11^. However, a meta-analysis reported that the prevalence of smoking physicians was high, approximately 21%, particularly in males^12^. Limited research has been conducted on the knowledge and attitudes toward ECs and HTPs among physicians. A mixed-methods systematic review showed that most healthcare professionals believe ECs are safer than CCs^13^. However, they harbor concerns regarding the short- and long-term safety of ECs^13^. Another systematic review indicated that physicians exhibit diverse opinions regarding the agreement of ECs for smoking cessation^14^. The majority of physicians demonstrated a deficiency in knowledge and confidence when engaging in conversations with patients about the safety and efficacy of ECs as alternatives for smoking cessation^14^. Thus, a proactive recommendation of ECs is not a common practice among most physicians. A cross-sectional study of 322 Turkish family physicians in 2019 revealed that merely 9.0% were aware of HTPs, and 83.9% expressed no discernible opinion on their usage^15^. A previous study focused on awareness and concerns regarding HTPs among Japanese physicians revealed that most physicians exhibited awareness of HTPs^16^. Furthermore, approximately half of them actively inquired about their patients’ HTP usage^16^. Nonetheless, the study also revealed a low level of concern regarding HTP addiction and regulatory policies among physicians^16^. However, the study had certain limitations, such as its limited sample size, primarily comprising participants aged <40 years, and the absence of inquiries regarding HTPs and ECs knowledge^16^.

To address the limitations arising from small sample sizes among young physicians and the inability to determine their proficiency pertaining to HTPs and ECs, this study aimed to assess: 1) the prevalence rates of HTP, EC, and CC usage, 2) sources of information and knowledge regarding HTPs and ECs; and 3) concerns regarding HTPs among Japanese physicians.

## METHODS

### Participants and procedures

This cross-sectional online survey was conducted 1–4 March 2021. Participants were recruited from the roster of Planned Research, a prominent Internet research firm in Japan with a database of 53000 physicians from diverse medical specialties. The sample size was calculated based on the population size (Japanese physicians n=97819, aged 24–39 years, 2020) and an acceptable margin of error (0.05) using Slovin’s formula^17^ to align with budgetary constraints. Invitations to participate were sent via email, and respondents gained access to the survey via an embedded web link. Inclusion criteria were physicians aged 24–39 years who practiced in Japan. Exclusion criteria were physicians who had already responded to the Japan Medical Association mail survey in 2020. Informed consent was obtained from all participants. The survey concluded after the predetermined target number was achieved. This study was approved by the Ethics Committee of Nihon University School of Medicine (Protocol Number: P20-27-0).

### Measures

The questionnaire was developed based on a previous survey conducted among Japanese physicians^16^ and a survey of Polish physicians’ awareness of electronic cigarettes^18^. Tobacco products were classified into CCs, cigars, chewing tobacco, snuff, HTPs, and ECs. To distinguish between CCs, HTPs, and ECs, the survey incorporated the most prevalent brands of HTPs and ECs. The questionnaire comprised three key domains: awareness, experience, and knowledge and concerns. Participants’ anonymity was maintained. Each participant was presented with at least four out of eight questions, and subsequent questions were based on their prior responses. The survey was designed to be completed within 7 minutes.

### Awareness of HTPs and ECs

Awareness of HTPs and ECs was assessed with the question: ‘Are you familiar with HTPs or ECs?’. The response options were: 1) Both HTPs and ECs; 2) HTPs only, but not ECs; 3) ECs only, but not HTPs; and 4) Neither of them. Participants who selected ‘Both’ or ‘HTPs only’ were classified as being aware of HTPs, while those who selected ‘Both’ or ‘ECs only’ were classified as being aware of ECs^16^.

### Smoking status

Current smoking status was classified into several categories: ‘Non-smokers (individuals who had never used tobacco products or abstained for the past month)’, ‘Dual smokers (used HTPs and ECs within the past month or smoked HTPs/cigarettes with other types in the past month)’, ‘Exclusive HTPs smokers (exclusively used HTPs in the past month)’, ‘Exclusive ECs smokers (exclusively used ECs in the past month)’, and ‘Other smokers (smoked CCs and other types; however, abstained from HTPs and ECs in the past month)’. Additionally, regarding knowledge of HTPs and ECs, and concerns associated with HTPs, individuals classified as former smokers were those who smoked at least once in the past month but were not currently smoking, while current smokers were defined as those who smoked within the past month, indicating ongoing smoking behavior. These groups constituted experienced smokers.

### Source of information regarding HTPs and ECs

The following nine options were used for sources of information on HTPs and ECs: 1) newspapers and stories; 2) television; 3) magazines; 4) SNSs or blogs; 5) tobacco company advertising; 6) acquaintances; 7) scientific literature; 8) medical portal sites for physicians; and 9) other. Participants could choose multiple options that applied to them.

### Knowledge of HTPs and ECs

Guided by the ‘Opinions and Recommendations on Heated Tobacco Products and Electronic Cigarettes’ outlined by the Japanese Respiratory Society^19^, ten questions were created. Regarding HTPs, the questions encompassed the following aspects: 1) recognition that HTPs were classified as ‘manufactured tobacco’ sanctioned by the ‘Tobacco Business Law’; 2) understanding that HTPs employed heated, processed leaf tobacco to generate aerosols; 3) awareness of the two HTPs variants: high-temperature and low-temperature heating types; 4) acknowledgment that HTP mainstream smoke contained harmful constituents, such as nicotine and carcinogens; 5) realization that HTP smokers’ sidestream smoke contained substances detrimental to health; and 6) recognition of reported instances of acute lung injury attributed to HTP usage. Regarding ECs, the questions probed the following dimensions: 1) comprehension that ECs heated a solution, termed e-liquid, to effectuate vaporization, with subsequent inhalation of the produced aerosol; 2) awareness that e-liquid may or may not contain nicotine; 3) knowledge that the manufacture and sale of ECs that contained nicotine were not sanctioned in Japan; and 4) recognition of the various additives and fragrances added to e-liquid, which could potentially result in the generation of deleterious substances. Scores of 1 or 0 were allocated for responses that indicated awareness and ignorance, respectively. Consequently, the comprehensive knowledge score ranged from 0 to 10 points. This score was employed as a continuous variable.

### Physicians’ concerns for HTPs

Physicians who exhibited awareness of HTPs were asked 13 questions concerning their individual apprehensions pertaining to HTPs. These concerns were categorized into three themes: 1) health risks, 2) addiction, and 3) regulation based on the questionnaires of previous studies^16,18^.

### Other variables

This survey collected personal demographic characteristics (e.g. sex, age group of 24–29 or 30–39 years) and professional characteristics (years of experience as a physician, workplace, and medical department).

### Statistical analyses

Participants’ demographics and the prevalence of the awareness of HTPs and ECs were examined. Subsequently, smoking status was examined and stratified according to sex. Primary sources of information on HTPs and ECs were elucidated. Knowledge levels that pertained to HTPs and ECs were compared with smoking and demographic attributes among participants who reported awareness of these products. Finally, concerns regarding the use of HTPs among experienced users were investigated. Categorical variables were reported as frequency and proportion with 95% confidence intervals (CIs). The chi-squared test was used to compare categorical variables. Weight adjustments were employed to ensure that the weighted proportions of participants across age groups and sexes corresponded with the national physician registration data, thereby furnishing nationally representative estimations as provided by the Ministry of Health, Labor and Welfare of Japan. Then, we conducted multivariate logistic regression analyses to explore participants’ knowledge of HTPs and ECs and their concerns about HTPs. In multivariate logistic regression models, we adjusted for smoking status, sex, age group, years of experience as a physician, workplace, and medical department. Total scores for knowledge of HTPs and ECs were performed using a one-way analysis of variance followed by Dunnett’s multiple comparisons *post hoc* test for each covariate. Statistical inferences were predicted on a significance threshold of p<0.05, and all tests were two-tailed. All statistical analyses were performed using Stata version 16.0 (Stata Corp).

## RESULTS

### Participants and awareness of HTPs and ECs

Table 1 shows the participants’ demographic characteristics. The survey was completed by 529 participants, of which 22.1% were females. Internal medicine (33.1%) was the predominant specialty, and 15.1% possessed a residency background. A sizeable majority (94.3%) were primarily employed in hospitals. In addition, 89.0% exhibited awareness of HTPs, and 71.3% were acquainted with ECs. Only 8.3% were unaware of both.

**Table 1:** Participant characteristics of a cross-sectional study for Japanese physicians aged 24–39 years, 1–4 March 2021 (N=529)

*Characteristics*	*n*	*%*
**Age** (years)		
24–29	214	40.5
30–39	315	59.6
**Sex**		
Female	117	22.1
Male	412	77.9
**Awareness of HTPs and ECs**		
HTPs and ECs	363	68.6
HTPs only	108	20.4
ECs only	14	2.7
Unknown	44	8.3
**Years of experience as a physician**		
1–2	81	15.3
3–5	165	31.2
6–10	160	30.3
11–15	123	23.3
**Workplace**		
General hospital	189	35.7
National public hospital	136	25.7
University hospital	174	32.9
Clinic	30	5.7
**Medical department**		
Internal medicine	175	33.1
Surgery	52	9.8
Orthopedics	36	6.8
Pediatrics	19	3.6
Gynecology	13	2.5
Dermatology	27	5.1
Urology	16	3.0
Psychiatry	29	5.5
Ophthalmology	17	3.2
Radiology	11	2.1
Otolaryngology	15	2.8
Emergency/anesthesiology	29	5.5
Other	10	1.9
Junior resident	80	15.1

HTPs: heated tobacco products. ECs: e-cigarettes.

### Smoking status

Table 2 presents the smoking status among young physicians according to sex. The estimated prevalence rates of exclusive CCs, ECs, HTPs, and dual usage were 4.3% (95% CI: 2.8–6.5), 2.9% (95% CI: 1.7–4.9), 1.9% (95% CI: 1.1–3.3), and 1.9% (95% CI: 1.0–3.6), respectively. Among male physicians, the prevalence of CC use (5.0%) was higher than that of exclusive EC (2.5%) and HTP (2.8%) use. Conversely, among female physicians, e-cigarette use (3.6%; 95% CI: 1.3–9.4) was most prevalent. Notably, no female participants reported HTP use.

**Table 2:** Current smoking status among Japanese physicians aged 24–39 years, 1–4 March 2021 (N=529)

*Smoking status*	*Total (N=529)*		*Male physicians (N=412)*		*Female physicians (N=117)*	
	*Weighted %*	*95% CI*	*Weighted %*	*95% CI*	*Weighted %*	*95 % CI*
Other smoker (exclusive)^a^	4.3	2.8–6.5	5.0	3.3–7.6	2.9	0.9–8.6
EC smoker (exclusive)	2.9	1.7–4.9	2.5	1.4–4.7	3.6	1.3–9.4
HTP smoker (exclusive)	1.9	1.1–3.3	2.8	1.6–4.9		
Dual smoker^b^	1.9	1.0–3.6	2.4	1.2–4.5	1.0	0.1–6.7
Non-smoker	89.0	86.0–91.5	87.3	83.7–90.3	92.6	85.8–96.3

Weight adjustments were employed to ensure that the weighted proportions of participants across age groups and sexes corresponded with the national physician registration data. HTPs: heated tobacco products. ECs: e-cigarettes.

a Other smokers are those who smoked cigarettes, except HTPs and ECs.

b Dual smokers are those who smoked cigarettes and HTPs, or cigarettes and ECs, or HTPs and ECs.

### Sources of information regarding HTPs and ECs

**Figure 1:**
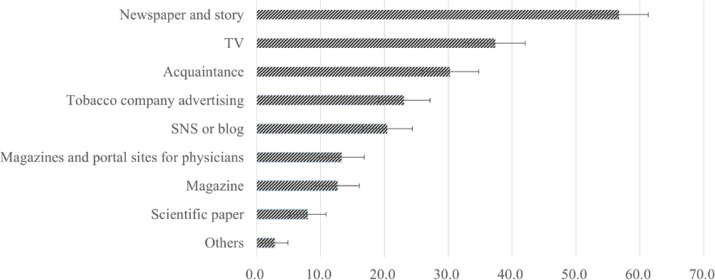
Sources of information regarding HTPs and ECs among Japanese physicians aged 24–39 years, 1–4 March 2021 (N=485)

Among participants with an awareness of HTPs and/or ECs, the primary sources of information were newspapers and stories (56.8%; 95% CI: 52.1–61.3), television (37.4%; 95% CI: 33.0–42.1), and acquaintances (30.3%; 95% CI: 26.2–34.8). Approximately 23.1% (95% CI: 19.5–27.1) reported they received information from tobacco company advertising, while 8.0% (95% CI: 5.9–10.9) gained insights from the scientific literature (Figure 1).

### Knowledge of HTPs and ECs

Table 3 and Supplementary file Table 1 present the data regarding knowledge of HTPs and ECs among participants aware of HTPs and/or ECs. A substantial proportion displayed familiarity that ‘HTPs mainstream smoke contained harmful ingredients, such as nicotine and carcinogens’ as well as ‘HTPs smokers’ sidestream smoke harbored detrimental substances’. Notably, current and former HTP users exhibited further accurate knowledge regarding HTPs compared to their counterparts. Conversely, non-smokers exhibited comparatively limited awareness, particularly regarding HTP types (33.2%) and the Tobacco Business Law (42.3%). Regarding participants with awareness of ECs, approximately half of the smokers demonstrated awareness that ‘ECs heated a solution called e-liquid to vaporize it and inhale the aerosol produced’. Current EC users displayed a further accurate understanding of HTPs, whereas non-smokers exhibited a diminished comprehension of ECs, particularly regarding the fact that ‘the manufacture and sale of ECs that contained nicotine was not approved in Japan’ (23.0%). Supplementary file Table 2 shows the participants’ scores reflective of the correct answers delineated by demographic characteristics. Those with prior experience with HTPs and/or ECs scored higher. Conversely, non-smokers had lower scores. Regarding specialties, physicians in otolaryngology, internal medicine, and dermatology demonstrated higher scores, whereas those in psychiatry and radiology had lower scores. There were no significant differences based on sex, age group, hospital affiliation, and years of experience.

**Table 3:** Knowledge of HTPs and ECs among Japanese physicians aged 24-39 years, 1–4 March 2021 (N=471)

*HTPs*	*Total (N=471)*		*Non-smoker (N=397)*		*Other smoker (N=26)*		*Former HTP smoker (N=26)*		*Current HTP smoker (N=22)*		p*
	*%*	*95% CI*	*%*	*95% CI*	*%*	*95% CI*	*%*	*95% CI*	*%*	*95 % CI*	
HTP is ‘manufactured tobacco’ approved by the Tobacco Business Law	46.9	40.8–50.2	41.3	36.3–46.5	66.0	45.0–82.1	62.5	41.1–79.9	83.7	62.5–94.0	<0.001
HTPs heat processed leaf tobacco to produce aerosols	48.7	43.6–53.8	51.4	46.7–56.1	62.5	41.7–79.6	77.5	56.5–90.1	64.3	43.3–80.9	0.024
HTPs are of two types: high-temperature and low-temperature heating type	32.3	27.8–37.3	38.3	33.8–42.9	61.6	41.0–78.8	86.8	65.2–95.8	73.2	53.4–86.6	<0.001
HTP smokers’ mainstream smoke contains harmful ingredients such as nicotine and carcinogens	75.4	70.7–79.5	75.6	71.3–79.5	82.8	60.8–93.7	78.6	56.7–91.2	69.7	48.0–85.2	0.757
HTP smokers’ sidestream smoke harbors detrimental substances	75.6	71.0–79.8	75.4	71.1–79.2	85.3	66.1–94.6	57.2	35.2–76.6	75.2	54.3–88.5	0.176
Acute lung injury due to the use of HTPs has been reported	56.6	51.4–61.6	59.6	54.8–64.1	73.2	52.9–86.9	73.6	51.3–88.0	84.0	63.8–94.0	0.012
***ECs***	***Total (N=377)***		***Nonsmoker (N=310)***		***Other smoker (N=22)***		***Former ECs smoker (N=22)***		***Current ECs smoker (N=23)***		p*
	***%***	***95% CI***	***%***	***95% CI***	***%***	***95% CI***	***%***	***95% CI***	***%***	***95 % CI***	
ECs heat a solution called e-liquid to vaporize it and inhale the aerosol produced	46.9	41.7–52.2	40.8	35.2–46.6	68.0	46.6–83.8	81.3	59.4–92.8	83.6	58.4–94.9	<0.001
E-liquid may or may not contain nicotine	40.9	35.8–46.2	35.9	30.5–41.7	65.5	44.4–81.9	58.6	36.9–77.5	73.5	48.4–89.1	<0.001
The manufacture and sale of ECs containing nicotine is not approved in Japan	28.1	23.6–31.1	23.0	18.5–28.3	49.8	30.7–68.9	56.9	35.3–76.1	53.1	29.2–75.7	<0.001
Various additives and fragrances are added to e-liquid, and heating it may generate harmful substances	45.7	40.5–51.0	43.8	38.2–49.7	40.6	23.7–60.0	69.1	47.3–84.8	57.0	32.3–78.7	0.102

HTPs: heated tobacco products. ECs: e-cigarettes. Excluding those who reported no awareness of HTPs or ECs.

* Calculated by the chi-squared test. Weight adjustments were employed to ensure that the weighted proportions of participants across age groups and sexes corresponded with the national physician registration data.

### Physicians’ concerns for HTPs

Table 4 and Supplementary file Table 3 present the concerns regarding HTPs among participants aware of HTPs. Overall, concerns across all the statements were most and least pronounced among non-smoking physicians and current or former HTP users, respectively, barring statements that addressed the lack of evidence regarding the long-term safety of HTP usage. Remarkably, 54.8% concurred with this assertion, which highlighted the absence of evidence. Furthermore, one-third expressed concerns regarding the long-term health implications of nicotine addiction. No current HTP users reported that ‘HTP use could inadvertently perpetuate smokers’ addiction’ or ‘HTP users could concurrently engage in dual usage alongside CCs’. Current users reported the lowest concerns regarding statements related to HTP marketing and advertising, especially for children and youth.

**Table 4:** Concerns regarding HTPs among Japanese physicians aged 24–39 years, 1–4 March 2021 (N=471)

*Concerns*	*Total (N=471)*		*Non-smoker (N=397)*		*Other smoker (N=26)*		*Former HTP smoker (N=26)*		*Current HTP smoker (N=22)*		p*
	*Weighted %*	*95% CI*	*Weighted %*	*95% CI*	*Weighted %*	*95% CI*	*Weighted %*	*95% CI*	*Weighted %*	*95% CI*	
**Health effects of HTPs**											
Lack of evidence regarding the long-term safety of the product	54.8	50.0–59.5	56.5	51.3–61.5	61.6	41.6–78.3	31.3	15.6–52.8	42.8	23.7–64.3	0.059
HTPs may cause acute lung injury	40.2	35.6–44.9	43.8	38.7–49.0	21.5	8.8–43.7	20.7	8.2–43.2	14.4	5.2–34.0	0.003
HTPs should be equated with electronic cigarettes	34.4	30.0–39.0	36.5	31.6–41.6	35.8	18.7–57.4	22.2	9.6–43.3	6.2	1.5–22.2	0.019
HTPs are misunderstood as being less harmful than cigarettes	26.1	22.1–30.6	28.8	24.3–33.8	9.9	2.2–34.8	14.7	4.7–37.6	5.1	0.7–28.6	0.029
HTPs are misunderstood as not causing passive smoking	29.3	25.1–33.8	32.9	28.1–38.0	19.8	7.8–41.6	4.1	0.6–24.0	0.0		<0.001
**Addictive potential of HTPs**											
Long-term health effects of nicotine addiction	30.1	25.9–34.7	32.8	28.1–37.9	18.7	6.9–41.6	13.1	4.0–35.6	11.3	3.5–31.0	0.033
HTP use may instead perpetuate smokers’ addiction	17.0	13.7–21.0	19.0	15.2–23.4	17.1	5.5–42.3	0.0		0.0		0.030
HTP user would be a dual user with cigarettes	8.5	6.2–11.7	9.2	6.6–12.7	7.2	1.0–36.6	6.6	1.6–23.8	0.0		0.575
**Regulation of HTPs**											
Virtual absence of regulatory controls by the government	29.0	24.8–33.6	32.1	27.4–37.2	14.3	4.4–37.8	10.7	2.6–34.7	8.2	1.9–28.6	0.014
Function as attractive starter products and a gateway to smoking for young non-smokers	48.5	43.8–53.3	54.2	49.0–59.3	17.0	6.1–39.5	6.6	1.6–23.8	24.5	10.1–48.6	<0.001
Marketing and advertising of HTPs, especially targeting children and youth	25.7	21.7–30.2	26.7	22.3–31.6	25.9	11.2–49.3	26.3	12.3–47.4	5.1	0.7–28.6	0.234
Become a ‘bridge product’ for use in places where smoking is prohibited	25.2	21.3–29.7	27.5	23.0–32.4	17.0	6.1–39.5	8.1	2.0–27.4	11.3	2.5–38.6	0.081
HTP advertising featuring celebrities vaping may make cigarette smoking glamorous again and ‘renormalize’ smoking	19.8	16.2–23.9	21.3	17.4–25.9	12.6	3.6–35.5	5.0	1.2–18.3	15.2	5.0–38.0	0.151

HTPs: heated tobacco products. ECs: e-cigarettes. Excluding those who reported no awareness of ECs.

* Calculated by the chi-squared test. Weight adjustments were employed to ensure that the weighted proportions of participants across age groups and sexes corresponded with the national physician registration data.

## DISCUSSION

This study is novel in that it represents the first comprehensive assessment of the knowledge and concerns associated with the use of HTPs and ECs among young physicians in Japan. The findings revealed that young male physicians preferred HTPs and ECs over CCs, whereas female physicians preferred ECs over both CCs and HTPs. In addition, newspapers and stories emerged as the primary source of information. Non-smoking physicians exhibited less knowledge concerning HTPs and ECs compared to their smoking counterparts. Furthermore, physicians’ smoking status had a significant influence on their concerns regarding HTPs.

Smoking prevalence among physicians demonstrated regional, sex-based, and age-based disparities. In this study, the prevalence of current smoking among male and female physicians was 12.7% and 7.4%, respectively. A systematic review that included studies from 48 countries found smoking prevalence rates of 29% and 12% among male and female physicians, respectively^20^. While the prevalence observed in this study was lower than the global average, it exceeded prior findings from nationwide surveys with Japanese physicians^16^. Furthermore, the prevalence of HTP and/or EC usage surpassed that of other tobacco products, consistent with the broader trend of younger individuals gravitating toward HTPs and ECs^20^. The reduced smoking prevalence among Japanese physicians is commendable. However, vigilance must be maintained regarding the potential future proliferation of HTPs.

Prior research on adults, including physicians, yielded limited insights into the primary sources of information on HTPs and ECs. Our findings differ somewhat from existing studies in other countries. For example, a cross-sectional survey that encompassed Korean physicians who specialized in lung cancer revealed that the predominant sources of EC information were the media and advertisements^21^. Furthermore, only 20% of the participants derived insights from professional sources such as scientific literature^21^. A study involving 399 medical students in Saudi Arabia found that social media, the Internet, and television advertisements served as their primary sources of EC information^22^. A cross-sectional study of 412 physicians in Poland indicated that the main sources of information about ECs were news stories or points about ECs^18^. Meanwhile, two cross-sectional surveys that targeted US adults identified Internet websites and social media accounts as prevalent sources^23^. A survey conducted among US physicians revealed that their primary recourse for accessing the latest research, updates in a particular disease area, and information pertinent to specific patient issues, is the Internet^24^. Despite the widespread use of the Internet, the Japanese general population still values traditional information sources such as newspapers and television, and may not widely use the Internet to obtain health-related information^25^. In addition, social media and TV programs supported by the tobacco industry indirectly promote HTPs in Japan^1,3^. Similarly, young Japanese physicians may still value traditional information sources and may not trust social media extensively to obtain information on HTPs and ECs. Alternatively, it is possible that young Japanese physicians have little interest in HTPs and ECs and, therefore, do not intentionally use specialized information sources. Future studies need to investigate how young Japanese physicians seek medical information.

This study highlights that even among physicians aware of HTPs and ECs, a deficiency of accurate knowledge persists – a trend mirrored in previous research. A cross-sectional study that involved 277 physicians, pharmacists, nurses, and public health practitioners in Japan reported that 62% lacked knowledge regarding HTPs^26^. A US-based study that encompassed physicians found that fewer physicians had detailed knowledge of ECs^27^. These findings emphasize the urgent need for additional information and training for physicians concerning HTPs and ECs. Given the widespread availability and popularity of HTPs in Japan, physicians encounter challenges in guiding patients on smoking cessation. Clear guidance elucidating the role of HTPs and ECs is essential for comprehensive awareness. Physicians express a desire for enhanced training and information to facilitate informed discussions on HTPs and ECs, addressing patient concerns with confidence and ease. This underscores the imperative for public educational campaigns targeting HTPs and ECs, disseminated through mass media channels such as newspapers, to foster heightened awareness and accurate comprehension among the general population.

Regarding HTPs, our findings broadly align with prior research focusing on Japanese physicians^16^. Notably, non-smokers consistently emerged as the most concerned demographic, whereas HTP users expressed the lowest levels of concern. Nevertheless, the current study diverges from earlier findings and indicates that HTP users exhibit lower levels of concern regarding HTP safety. This shift may be indicative of age-related differences in the results. Intriguingly, other tobacco users showed the highest levels of concern regarding HTP safety. This suggests that individuals engaged in other tobacco products harbor reservations regarding a transition to HTPs owing to their heightened apprehensions surrounding HTP safety.

### Strengths and limitations

This study has several important strengths. First, the findings of this study are anticipated to furnish crucial insights into smoking cessation education for healthcare practitioners, given that limited data exist regarding the perspectives of healthcare professionals concerning HTPs. Second, our target sample is concentrated within the demographic wherein HTPs and ECs have garnered popularity.

Although the present study reveals important findings, it has certain limitations. First, the survey was conducted through a panel managed by a private monitoring company. Thus, selection bias, such as response bias, may have occurred as the participants were not randomly selected. Consequently, the generalizability of these findings may be limited. Second, the study design was cross-sectional, which precluded the establishment of causal relationships. Third, social desirability bias may have led to an underestimation of smoking prevalence; physicians, particularly females in Japan, may not have wished to reveal their smoking behavior^28^. Fourth, confounding factors such as mental health may not have been adjusted for. Fifth, it may not be possible to generalize to other countries because the prevalence of ECs and HTPs varies by country.

## CONCLUSIONS

The findings of this study indicate that non-smoking was the predominant disposition among young physicians in Japan. Nonetheless, there was a preference for HTP use among young male physicians. Smoking status influenced knowledge regarding HTPs and ECs. Furthermore, non-smokers exhibited diminished awareness of these products. The primary sources of information for young physicians were traditional news sources. Given that physicians often serve as the primary source of medical guidance and are entrusted with encouraging smoking cessation among their patients, concentrated efforts are required to equip them with the requisite knowledge and understanding of HTPs and ECs, irrespective of their medical specialty. Thus, it is imperative to adapt efficacious campaigns to ensure widespread dissemination of accurate knowledge concerning HTPs and ECs. Future investigations into the evolving attitudes of Japanese physicians toward HTPs and ECs are required to assess smoking cessation strategies in Japan.

## Data Availability

The data supporting this research are available from the authors on reasonable request.
