# Supplementary material for: Prevalence, knowledge, and concerns regarding the use of heated tobacco products and electronic cigarettes among young Japanese physicians
Source: Tob Induc Dis. 2024 Feb 16;22:10.18332/tid/178508. doi: 10.18332/tid/178508 (PMC10870344; doi:10.18332/tid/178508)
Supplement: Supplementary file 1 [file TID-22-42-s1.pdf]

Supplementary Materials

Supplementary Table 1. Odds ratios regarding knowledge of HTPs and ECs among Japanese physicians aged 24-39 years, 2021 (N=377-471)

HTPs	Other smoker (N=26)		p-value	Former HTP smoker (N=26)		p-value	Current HTP smoker (N=22)		p-value
	aOR	95%CI		aOR	95%CI		aOR	95%CI	
HTP is "manufactured tobacco" approved by the "Tobacco Business Law."	2.65	1.09-6.46	0.032	2.28	0.94-5.53	0.068	6.84	2.14-21.80	0.001
HTP heats processed leaf tobacco to produce aerosols.	1.91	0.79-4.65	0.152	1.69	0.70-4.08	0.242	3.58	1.33-9.65	0.012
HTPs are of two types: high-temperature and low-temperature heating type.	6.03	2.42-15.03	< 0.001	3.05	1.25-7.47	0.015	14.72	3.99-54.25	< 0.001
HTP aerosols contain harmful ingredients such as nicotine and carcinogens.	0.80	0.30-2.18	0.665	1.57	0.48-5.10	0.454	1.22	0.43-3.46	0.715
HTP smokers' breath contains harmful ingredients.	1.08	0.41-2.84	0.883	1.86	0.61-5.62	0.272	0.43	0.17-1.09	0.075
Acute lung injury due to the use of HTPs has been reported.	3.89	1.26-12.03	0.018	2.05	0.79-5.28	0.138	1.95	0.71-5.34	0.192
**ECs**	**Other smoker (N=22)**		**p-value**	**Former EC smoker (N=22)**		**p-value**	**Current EC smoker (N=23)**		**p-value**
	OR	95%CI		OR	95%CI		OR	95%CI	
ECs heat a solution called e-liquid to vaporize it and inhale the aerosol produced.	3.93	1.40-10.99	0.009	3.71	1.26-10.89	0.017	6.78	2.14-21.45	0.001
E-Liquid may or may not contain nicotine.	4.37	1.66-11.54	0.003	5.01	1.63-15.40	0.005	2.06	0.82-5.14	0.122
The manufacture and sale of ECs containing nicotine is not approved in Japan.	2.45	0.96-6.24	0.061	4.83	1.84-12.68	0.001	4.22	1.65-10.82	0.003

Various additives and fragrances are added to the E-liquid, and heating it may generate harmful substances.	1.99	0.80-4.96	0.141	1.16	0.46-2.90	0.756	1.47	0.58-3.76	0.419

HTPs: Heated tobacco products; ECs: e-cigarettes.

Excluding those who reported no awareness of HTPs or ECs.

Odds ratios were performed by logistic regression adjusted for smoking status, sex, age group, years of experience as a physician, workplace, and medical department.

Reference category was non-smoker (N=377-397)

Supplementary Table 2. Scores for knowledge of HTPs and ECs by participant demographics among Japanese physicians aged 24-39 years, 2020 (N=363).

		N	Score	95%CI			p-value
Age group							
	24-29	145	5.71	5.19	-	6.23	0.891
	30-39	218	5.34	4.92	-	5.76	
Sex							
	Female	64	5.28	4.52	-	6.04	0.638
	Male	299	5.53	5.17	-	5.89	
Smoking experience							
	Non-smoker	288	5.10	4.74	-	5.46	ref
	Other smoker	22	6.64	5.35	-	7.93	0.080
	HTPs (exclusive)	11	**7.82**	**5.99**	**-**	**9.64**	**0.015**
	ECs (exclusive)	13	7.00	5.32	-	8.68	0.097
	HTPs and ECs	29	**6.90**	**5.77**	**-**	**8.02**	**0.010**
Years of experience as a physician							
	1-2 years	59	5.53	4.71	-	6.34	
	3-5 years	107	5.62	5.01	-	6.22	0.996
	6-10 years	116	5.26	4.68	-	5.84	0.914
	11-15 years	81	5.62	4.92	-	6.31	0.997
Workplace							
	General hospital	124	5.44	4.88	-	5.99	
	National public hospital	96	5.30	4.67	-	5.94	0.980
	University hospital	119	5.43	4.86	-	6.00	1.000
	Clinic	24	6.79	5.53	-	8.06	0.134
Medical department							
	Internal medicine	119	6.08	5.51	-	6.64	
	Surgery	37	4.57	3.56	-	5.58	0.092
	Orthopedics	28	4.79	3.62	-	5.95	0.334
	Pediatrics	14	5.14	3.50	-	6.79	0.946
	Gynecology	8	5.00	2.82	-	7.18	0.977
	Dermatology	20	6.15	4.77	-	7.53	1.000
	Urology	11	5.64	3.78	-	7.49	1.000
	Psychiatry	14	3.71	2.07	-	5.36	0.069
	Ophthalmology	11	5.55	3.69	-	7.40	1.000
	Radiology	6	4.33	1.82	-	6.85	0.795
	Otolaryngology	9	7.33	5.28	-	9.39	0.900
	Emergency/Anesthesiology	24	4.79	3.53	-	6.05	0.416
	Other	6	5.67	3.15	-	8.18	1.000
	Junior residents	56	5.63	4.80	-	6.45	0.985

HTPs: Heated tobacco products. ECs: e-cigarettesExcluding those who reported no awareness of HTPs and ECs.

Total scores for knowledge of HTPs and ECs were performed using a one-way analysis of variance followed by Dunnett's multiple comparisons post hoc test for each covariate.

Supplementary Table 3. Odds ratios regarding concerns about HTPs among Japanese physicians aged 24-39 years, 2021 (N=471)

	Other smoker (N=26)		p-value	Former HTP smoker (N=26)		p-value	Current HTP smoker (N=22)		p-value
	aOR	95%CI		aOR	95%CI		aOR	95%CI	
Health effects of HTPs									
Lack of evidence regarding the long-term safety of the product.	1.23	0.53-2.85	0.635	0.35	0.14-0.89	0.027	0.59	0.24-1.45	0.252
HTPs may cause acute lung injury.	0.38	0.13-1.08	0.070	0.33	0.11-0.96	0.042	0.23	0.07-0.74	0.014
HTPs should be equated with electronic cigarettes.	1.00	0.42-2.34	0.993	0.56	0.20-1.58	0.271	0.11	0.02-0.52	0.005
HTPs are misunderstood as being less harmful than cigarettes.	0.27	0.05-1.34	0.108	0.45	0.12-1.60	0.215	0.12	0.02-0.96	0.045
HTPs are misunderstood as not causing passive smoking.	0.51	0.17-1.59	0.247	0.09	0.01-0.63	0.016	–	–	–
Addictive potential of HTPs									
Long-term health effects of nicotine addiction.	0.51	0.16-1.63	0.257	0.30	0.08-1.16	0.081	0.25	0.07-0.98	0.047
HTP use may instead perpetuate smokers' addiction.	0.89	0.27-2.99	0.851	–	–	–	–	–	–
HTP user would be dual user with cigarettes.	0.69	0.08-5.81	0.734	0.75	0.16-3.57	0.716	–	–	–
Regulation of HTPs									
Virtual absence of regulatory controls by the government.	0.37	0.10-1.36	0.135	0.25	0.06-1.14	0.074	0.19	0.04-0.88	0.034
Function as attractive starter products and a gateway to smoking for young nonsmokers.	0.17	0.05-0.56	0.004	0.06	0.01-0.28	< 0.001	0.26	0.09-0.80	0.019
Marketing and advertising of HTPs, especially targeting children and youth.	0.99	0.37-2.66	0.992	0.99	0.35-2.78	0.979	0.13	0.02-1.04	0.054
Become a "bridge product" for use in places where smoking is prohibited.	0.55	0.16-1.88	0.342	0.24	0.05-1.07	0.061	0.31	0.07-1.42	0.133

HTP advertising featuring celebrities vaping may make cigarette smoking glamorous again and "renormalize" smoking.	0.53	0.13-2.14	0.368	0.19	0.04-0.85	0.03	0.63	0.18-2.20	0.466

HTPs: Heated tobacco products; ECs: e-cigarettes.

Excluding those who reported no awareness of ECs. Odds ratios were performed by logistic regression adjusted for smoking status, sex, age group, years of experience as a physician, workplace, and medical department. Reference category was non-smoker (N=397).

© 2024 Otsuka Y. et al.
